# Needle in a Whey-Stack: PhRACS as a Discovery Tool for Unknown Phage-Host Combinations

**DOI:** 10.1128/mbio.03334-21

**Published:** 2022-01-04

**Authors:** Eoghan Casey, Brian McDonnell, Kelsey White, Panagiota Stamou, Tadhg Crowley, Ian O’Neill, Katherine Lavelle, Stephen Hayes, Gabriele A. Lugli, Silvia Arboleya, Kieran James, Marco Ventura, Ines Martinez, Miguel Gueimonde, Fabio dal Bello, Ken Nally, Jennifer Mahony, Douwe van Sinderen

**Affiliations:** a School of Microbiology, University College Cork, Cork, Ireland; b APC Microbiome Ireland, University College Cork, Cork, Ireland; c Flow Cytometry Platform, APC Microbiome Ireland, University College Cork, Cork, Ireland; d Laboratory of Probiogenomics, Department of Chemistry, Life Sciences and Environmental Sustainability, University of Parma, Parma, Italy; e Microbiota, Food and Health Group, Department of Biochemistry and Microbiology of Dairy Products, Institutode Productos Lácteos de Asturias, Consejo Superior de Investigaciones Científicas, Villaviciosa, Spain; f Sacco Srl, Cadorago (Co), Italy; Universidade de Sao Paulo

**Keywords:** metagenome, virome, phageome, cytometry, fluorescent, RBP, fecal, whey, fluorescence

## Abstract

The field of metagenomics has rapidly expanded to become the go-to method for complex microbial community analyses. However, there is currently no straightforward route from metagenomics to traditional culture-based methods of strain isolation, particularly in (bacterio)phage biology, leading to an investigative bottleneck. Here, we describe a method that exploits specific phage receptor binding protein (RBP)-host cell surface receptor interaction enabling isolation of phage-host combinations from an environmental sample. The method was successfully applied to two complex sample types—a dairy-derived whey sample and an infant fecal sample, enabling retrieval of specific and culturable phage hosts.

## INTRODUCTION

For over a century, culture-dependent methods have been employed as the primary method for (bacterio)phage detection, isolation, and propagation (1–3). The recent proliferation of virome/phageome studies (4–7) has supplanted such traditional methods of phage identification and has led to a vast collection of presumed phage sequences associated with unknown hosts. However, functional characterization of phages identified by a virome approach will (in most cases) require identification of a permissive host with subsequent propagation to high virion levels.

Following virome/phageome sequence read generation, bioinformatic approaches may predict bacterial hosts of putative phages associated with an identified contiguous sequence (contig), though such predictions are not necessarily robust or specific (8). Host predictions are mainly based on (i) detection of genetic identity with putative hosts, where phages may have acquired genetic elements from a previous host (success rate in host species prediction for 820 phages, ∼38.5%) (8), (ii) CRISPR spacer analysis (success rate, ∼21.3%) (8), and (iii) phage and host kmer profile analysis, i.e., an assessment of viral usage of oligonucleotide strings of a specific length, profiles of which tend to shift toward those of the host over time (success rate, ∼17.2%, using 4-mer analysis [8]). A recent advancement in kmer analysis (HostPhinder), profiling 16-mers of phages and sequenced hosts, showed a substantially improved prediction of host species and genus at 74% and 81%, respectively, of analyzed phages (*n* = 2,196) (9). However, these predictions provide information at the genus level (or at best the species level) but cannot identify the specific strain on which target bacteriophages may propagate.

Further methods are available to physically link unpropagated phages to their hosts, including phageFISH, though this requires genome sequences of both for probe design (10). Viral tagging allows identification of phages that can infect a target host by means of fluorescent labeling of phages using a DNA stain and subsequent fluorescence-activated cell sorting (FACS) (11). Limiting the applicability of this method is a requirement for a pure culture and the ability to isolate phages infecting only one known host.

The first stage of phage infection requires a host-encoded receptor(s) being bound by a phage-encoded receptor binding protein (RBP) (12), a highly specific interaction (13). This is illustrated in detail by two phages, TP901-1 and Tuc2009, both belonging to the P335 phage group infecting Lactococcus cremoris. In TP901-1, the RBP is represented by the BppU protein which assembles as a trimer that is connected to three BppL proteins, forming a tripod (14, 15). In Tuc2009, a third (accessory) protein (“BppA”) is incorporated into this tripod complex (15, 16). These phages recognize distinct carbohydrate moieties on the cell surfaces of their respective hosts (13) due to C-terminal divergence in BppL (16).

Previous studies have used fluorescently labeled RBPs to show specific receptor binding activity in several genera (17–20) and have been proposed as a method for specific identification of clinically relevant bacterial species (21, 22). Herein, we present an integrated method capable of overcoming limitations of *in silico* phage-host prediction methods while addressing discontinuity between metagenomics and culture-based techniques. In a significant prelation, phage RBP-activated cell sorting (PhRACS) exploits specific RBP-host receptor interaction in order to link the RBP sequence of a given virome-derived, phage-associated contig to its corresponding receptor-containing host, and to enable isolation of both from a variety of ecological niches.

## RESULTS AND DISCUSSION

### Proof of concept i: GFP-RBP construction and known host labeling.

**FIG 1:**
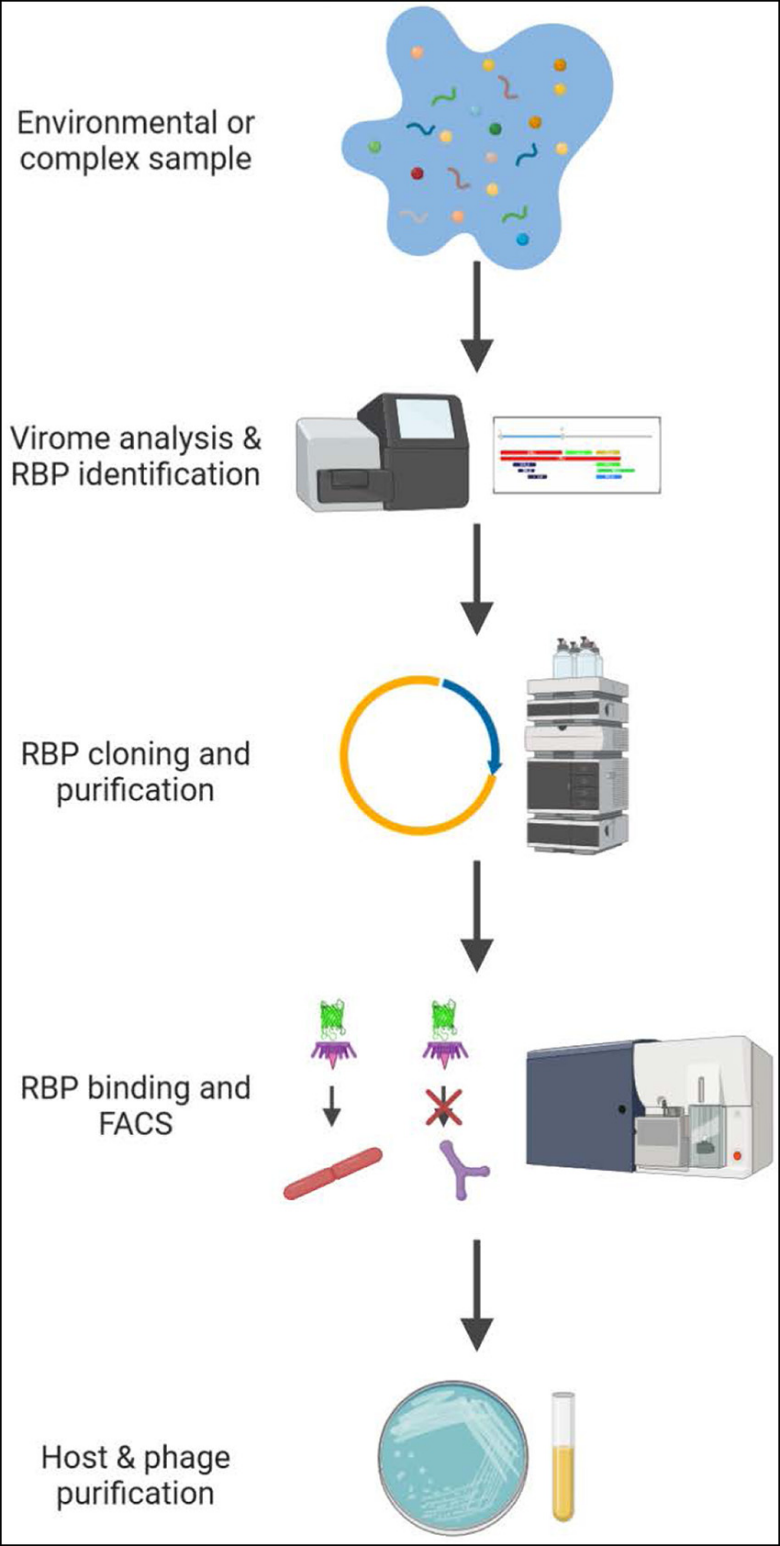
PhRACS workflow. Virome analysis is first performed on a complex sample, and putative RBP nucleotide sequences are identified. RBP-encoding genes are then cloned on a suitable plasmid and expressed. Incubation of GFP-tagged RBPs with the processed sample then allows isolation of the phage host from the complex sample by FACS.

To investigate the validity of the PhRACS methodology (Fig. 1), two established phage-host combinations, Tuc2009/L. cremoris UC509.9 and TP901-1/*L*. *cremoris* 3107 (Table 1), were employed to specifically label a single host using fluorescently tagged RBP of its infecting phage.

**TABLE 1:** Bacterial strains and plasmids used in this study

Bacterial strain or plasmid	Relevant characteristic(s)	Reference
Bacterial strains		
L. lactis		
NZ9000	MG1363 containing *nisRK* genes	64
3107	Host for TP901-1	65
UC509.9	Host for Tuc2009	66
SC34	Host for BB4_2	This study
*Leuconostoc*		
LMDS5	Host for LMD_1_2	This study
Escherichia coli		
BL21 (DE3)	Protein expression strain	New England Biolabs
Undefined starter culture		
MUO12		This study

Plasmids		
pNZ8048	Standard L. lactis expression vector, Cm^r^, nisin inducible	64
pGFP8048	pNZ8048 derivative harboring GFP, contains a His tag cloned in frame	This study
pNZ44	Standard L. lactis expression vector, Cm^r^	67
pNZ8048-His-GFP-UAL_Tuc2009_	pNZ8048 encoding Tuc2009 *bppU*, *bppA*, *bppL* as an operon with an N-terminally His-tagged GFP fused to the N terminus	This study
pNZ8048-His-GFP-UL_TP901-1_	pNZ8048 encoding TP901-1 *bppU* and *bppL* as an operon with an N-terminally His-tagged GFP fused to the N terminus	This study
pGFP8048-His-GFP-RBP_LMD_	pGFP8048 encoding ΦLMD_1_2 *orf22*	This study
pHTP9-423phi1Rv1	pHTP9 encoding 423phi1Rv1 conformation	This study
pHTP9-RBP_BB4_2_	pHTP9 encoding BB4_2*_orf04_*	This study

**FIG 2:**
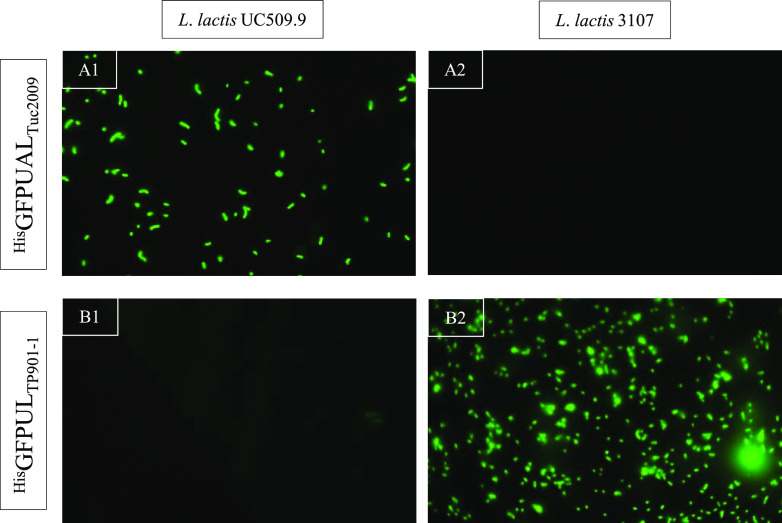
(A1) Interaction between recombinant ^His^GFP-UAL_2009_ and Tuc2009 host *L*. *cremoris* UC509.9. (A2) Interaction between recombinant ^His^GFP-UAL_2009_ and Tuc2009 nonhost *L*. *cremoris* 3107. (B1) Interaction between recombinant ^His^GFP-UL_TP901_ and TP901-1 nonhost *L*. *cremoris* UC509.9. (B2) Interaction between recombinant ^His^GFP-UL_TP901_ and TP901-1 host *L*. *cremoris* 3107.

His-tagged, green fluorescent protein (GFP)-RBP fusion complexes were constructed for each phage (22) as described in Materials and Methods and Tables 1 and 2. These complexes consisted of the full receptor binding protein assemblies (called tripods) of each phage (23), designated here as ^His^GFP-UAL_Tuc2009_ and ^His^GFP-UL_TP901-1_. Following purification, tripods were incubated with host or nonhost cells, and their association with cell surfaces was assessed by fluorescence confocal microscopy. In this way, qualitative analysis of the affinity of this RBP-receptor interaction was performed, as previously described for Lactobacillus casei and Listeria monocytogenes (18, 19). Specific binding to susceptible hosts was observed in each phage-host combination, i.e., ^His^GFP-UAL_Tuc2009_ exclusively binds to L. lactis UC509.9 cells (Fig. 2, panel A1), whereas ^His^GFP-UL_TP901-1_ specifically interacts with L. lactis 3107 cells (Fig. 2, panel B2), thus establishing that GFP-tagged, phage-derived RBPs can bind specifically to susceptible host cell surfaces.

**TABLE 2:** Oligonucleotides used in this study

Oligonucleotide	Sequence (5′-3′)^*a*^	Comment^*b*^	Restriction enzyme^*c*^
GFPFwHis	AGCAGCGCATGC**GTCACCATCATCATCATCAT**AGTAAAGGAGAAGAACTTTTC	F primer of gfp+ flanked by a His tag	SphI
GFPRevlinkBppU	TATAAAATGTTCTGTCAT*ACCAGAACCACCACCAGAACCACC*TTTGTAGAGCTCATCCAT	R primer of gfp+ flanked by a linker and region complementary to BppU N terminus	
BppUF	GGTTCTGGTATGACAGAACATTTTATA	F primer BppL Tuc2009/TP901-1	
BppLTucR	AGCAGCACTAGTCTAATTCCGATAAAGTTTTAC	R primer of BppL Tuc2009	SpeI
BppLTPR	AGCAGCACTAGTTTAATCTATTGGATATGTACC	R primer of BppL TP901-1	SpeI
GFP8048F	AGCAGCGCATGCGT**CACCATCATCATCATCAT**AGTAAAGGAGAAGAACTTTTC	F primer of gfp+ flnked by a His tag	SphI
GFP8048R	AGCAGCACTAGTGGTACCGATATCGAATTCACC*AGAACCACCACCAGAACCACC*TTTGTAGAGCTCATCCAT	R primer of gfp+ flanked by flexible linker with MCS (EcoRI, EcoRV, KpnI, SpeI, XbaI)	SpeI
LMD_22F	AAAAAAGAATTCACATTAGCAAATACAGAATTAGTAT	F primer LMD_1_22	EcoRI
LMD_22R	AAAAAAGGTACCTTAGTCTGGAGTTGCGCTTAACCAC	R primer LMD_1_22	KpnI
GFP423phi1F	*TCAGCAAGGGCTGAGGG*CAACAAATCTGCTTGTAAC	F primer targeting 423phi1_Rv1 flanked by pHTP9-specific overhang	NA
GFP423phi1R	*TCAGCGGAAGCTGAG*GCTACGCGGTAATCCAACAGCC	R primer targeting 423phi1_Rv1 flanked by pHTP9-specific overhang	NA
BB4-RBPF	*TCAGCAAGGGCTGAGG*ATGACAATTAAAAAATTCACGTT	F primer BB4_2_04 flanked by pHTP9-specific overhang	NA
BB4-RBPR	*TCAGCGGAAGCTGAGG*CTAATTTATACGTTTCCAAATAT	R primer BB4_2_04 flanked by pHTP9-specific overhang	NA

a Restriction sites are underlined. Linker and overhang sequences are italicized. His tag sequences are indicated in boldface type.

b MCS, multiple cloning sites.

c NA, not available.

### Proof of concept ii: Host retrieval by FACS.

The potential for host isolation PhRACS was then investigated. Two pooled cultures were generated, consisting of (i) Lactococcus lactis 3107 wild type and chloramphenicol-resistant L. lactis UC509.9::pNZ44 (pool A; for assessment using ^His^GFP-UAL_Tuc2009_) and (ii) chloramphenicol-resistant L. lactis 3107::pNZ44 and L. lactis UC509.9 wild type (pool B; for assessment using ^His^GFP-UL_TP901-1_).

Incubation of ^His^GFP-UAL_Tuc2009_ with pool A resulted in labeling of 32.2% of cells (see Fig. S1, panel A1, in the supplemental material), whereas plate counts of cells prior to incubation (Fig. S1, panel B, row 1) indicated a GFP-positive population of 56.5%. This discrepancy is likely due to the gating strategy employed, where a significant portion of the GFP-positive population was ungated (i.e., unaccounted for) due to its suboptimal purity (Fig. S1, panel A1; indicated by arrow). In total, 252,480 events (i.e., GFP-positive entities) were segregated into GM17 plus chloramphenicol. Resultant cultures were assessed for sensitivity to Tuc2009 by standard spot assay, with such sensitivity confirmed by a clearing zone on the bacterial lawn (Fig. S1, panel C1), establishing the ability to specifically retrieve viable host GFP-RBP-labeled cells by FACS.

10.1128/mbio.03334-21.1 FIG S1 (A) Dot plot analysis of mixed culture pools A and B. Pool A (A1) was labeled with ^His^GFP-UAL_Tuc2009_, whereas pool B (A2) (and subsequent dilutions of same; A3 to A9) were labeled with ^His^GFP-UL_TP901-1_. (B) Sample composition (CFU/mL) of mixed cultures analyzed by FACS, expected and actual percent GFP-positive events, and total number of events sorted. (C) Confirmation of recovery of host by spot assay with Tuc2009 (C1) and TP901-1 (C2 to C9), where visible zones of lysis indicate successful infection. Download FIG S1, EPS file, 2.3 MB. Copyright © 2022 Casey et al. 2022 Casey et al. https://creativecommons.org/licenses/by/4.0/ This content is distributed under the terms of the Creative Commons Attribution 4.0 International license.

Host retrieval was similarly demonstrated using ^His^GFP-UL_TP901-1_ following incubation with pool B (40.8% GFP positive, 39.3% expected positive; Fig. S1, panel B, row 2]) indicating that the GFP-tripod complex specifically binds (and thereby labels) hosts among a mixed population in both cases (Fig. S1, panel A2, and panel B, row 2). Sorted cells were collected and showed sensitivity to TP901-1 in the same manner as observed for Tuc2009 (Fig. S1, panel C2).

### Proof of concept iii: Limit of host retrieval.

To further assess the specificity of this interaction and to define the limit of host retrieval, serial dilutions of a fresh overnight (o/n) culture of 3107::pNZ44 to a theoretical target concentration of 1 CFU/mL were generated. These dilutions were mixed with nontarget cells (UC509.9) in various ratios (as per Fig. S1, panel B, rows 2 to 9) for assessment using ^GFP^UL_TP901-1_. As expected, the observed percentage of target GFP-positive population decreases to 0.066 (Fig. S1, panel A5; Fig. S1, panel B, row 5). However, nonspecific background fluorescence at approximately 0.02% to 0.04% remained in samples with the lowest levels of 3107::pNZ44 target cells (Fig. S1, panel B, rows 6 to 9). Despite this, cell sorting below this limit should theoretically still occur, and indeed retrieval of culturable target hosts at all tested target-nontarget cell ratios was demonstrated by subsequent reinfection using TP901-1 (Fig. S1, panels C3 to C9), despite the presence of background levels of fluorescence (Fig. S1, panel B, rows 6 to 9).

### Phageome analysis i: Dairy niche.

To assess whether PhRACS can retrieve an unknown phage-host combination from an industrial ecological niche, a dairy-derived whey sample was employed. Undefined mesophilic starter cultures used in cheese production represent complex communities composed of a large number of lactococcal strains with additional *Leuconostoc* species (24).

Phageome sequencing was first performed on a whey sample derived from a quark manufactured by an undefined mesophilic culture (termed MUO12). Assignment of taxonomic lineage to obtained sequence reads revealed a predicted *Leuconostoc* phage read abundance of approximately 21.6% within the sample. Following sequence assembly, a contig was identified that exhibited high nucleotide identity (98% across 97% of the contig) to *Leuconostoc* phage ΦLMD_1_2, which has been reported to infect Leuconostoc mesenteroides and Leuconostoc pseudomesenteroides strains (25). Kmer analysis of ΦLMD_1_2 using HostPhinder predicted *Ln. pseudomesenteroides* as the host for this phage (coverage, 1.4e−01) (9), and this contig was selected for further analysis.

**FIG 3:**
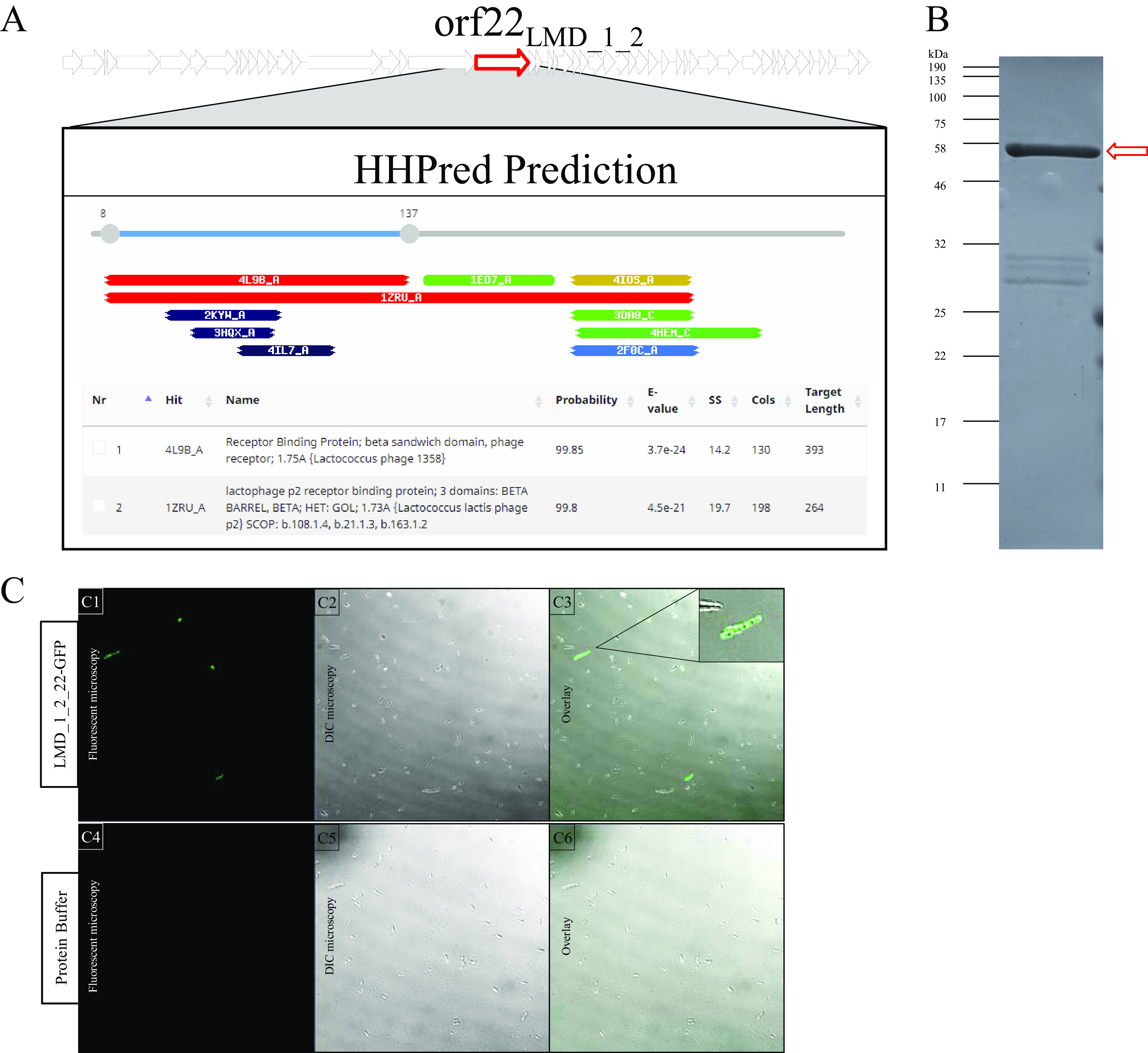
(A) HHpred analysis of *orf22*_LMD_1_2_, a putative RBP, displaying similarity to lactococcal phages 1358 and p2 RBPs. (B) Sodium dodecyl sulfate-polyacrylamide gel electrophoresis (SDS-PAGE) analysis of ^His^GFP-RBP_LMD_ fusion protein (indicated by the red arrow). (C) Putative phage-susceptible hosts within an undefined culture specifically labeled by putative GFP-tagged putative RBPs (C1 to C3), visualized using fluorescence microscopy. Protein buffer (C4 to C6) was employed as a control. DIC, differential interference contrast.

HHpred (26), which has been utilized to identify RBPs of phages infecting various genera (18, 19, 27–31), was employed to interrogate the gene products of ΦLMD_1_2 to identify the RBP-encoding gene of this phage. Analysis of the product of *orf22*_LMD_1_2_ revealed structural similarity to RBPs of lactococcal phages 1358 (probability = 99.85%) and p2 (probability = 99.8%) (Fig. 3, panel A) denoting this as the most likely candidate, and this gene was selected for cloning.

To test the robustness of the method, a second phageome analysis was performed on a whey sample from a separate MUO12-based fermentation. Here, most (90.6%) reads were predicted to originate from multiple L. lactis phages, all belonging to the *Skunavirus* (previously 936) phage group. Following sequence assembly, a contig (termed BB4_2) containing a partial phage genome was identified that exhibited high nucleotide identity (96% across 73% of the contig) with L. lactis phage 645 (32), with *orf04_BB4_2_* being identified as the likely RBP-encoding gene of this phage. Aligning ORF04*_BB4_2_* against previously sequenced 936 phage RBPs revealed it to be a member of the group III RBPs which are known to bind to either cell wall polysaccharide (CWPS) type B or C lactococcal hosts (33).

### Host retrieval i: Dairy niche.

Cloning of *orf22*_LMD_1_2_ into pGFP8048 allowed production of an ∼60-kDa protein representing the RBP of phage LMD_1_2 (Fig. 3, panel B), which was then purified (to give ^His^GFP-RBP_LMD_) and assessed for its ability to bind selectively to its cognate host cell receptor in a mixed starter culture. To achieve this, the whole L. lactis and *Leuconostoc* complements of the commercial starter were separately cultivated in selective media, and these cultures were pooled. ^His^GFP-RBP_LMD_ (or protein storage buffer [PB] as a negative control) was then added to this pooled culture as described in Materials and Methods. As expected, fluorescence microscopy revealed specific labeling of a subpopulation of the mixed culture (Fig. 3, panels C1 to C3), while the culture containing control remained unlabeled (Fig. 3, panels C4 to C6).

**FIG 4:**
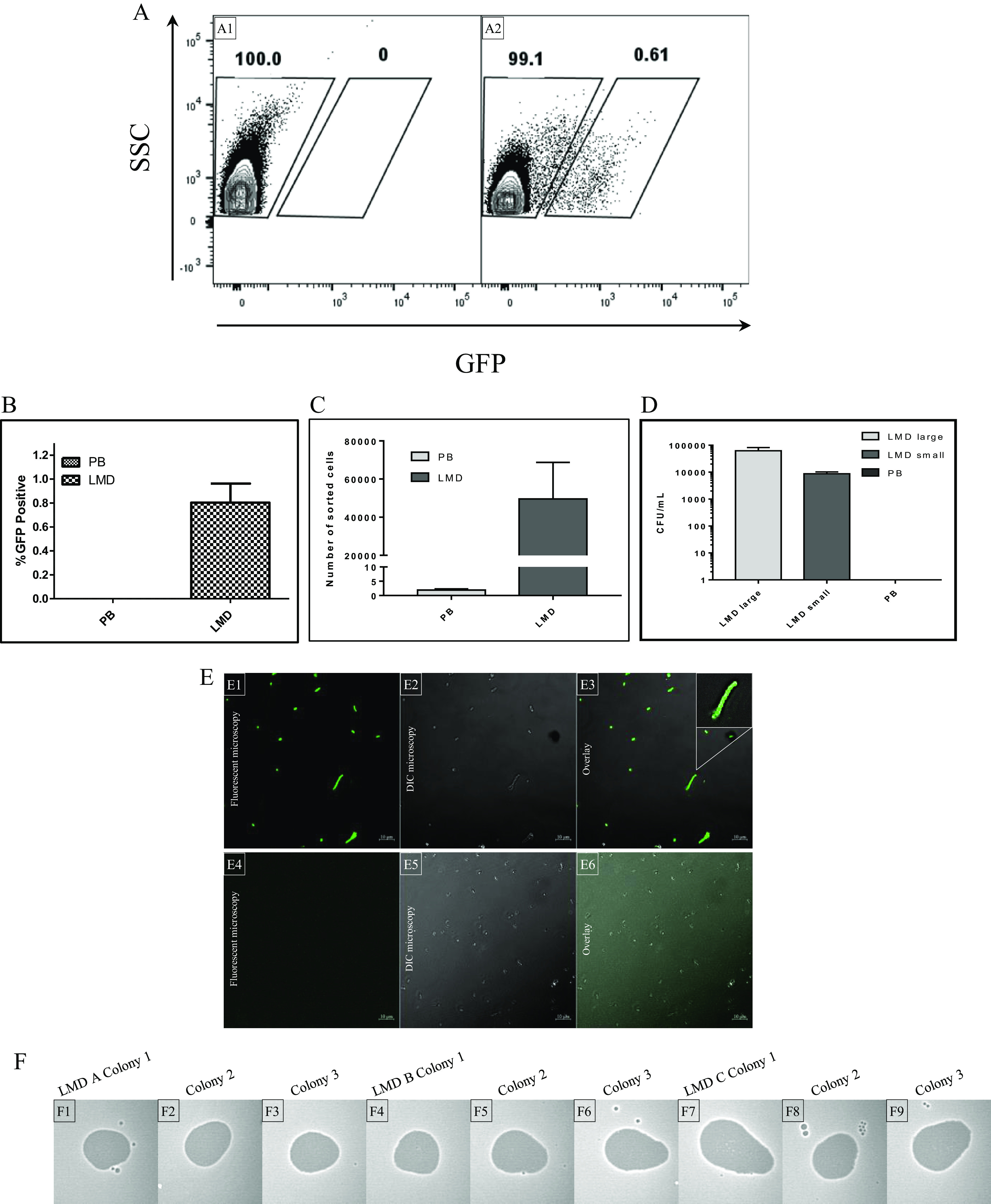
(A) Dot plot analysis of undefined culture MUO12 labeled with protein buffer (PB) (A1) and ^His^GFP-RBP_LMD_ (A2). SSC, side scatter. (B) Proportion of GFP-positive events expressed as a percentage of total events processed. (C) Number of events sorted using gating strategy targeting GFP-positive cells evident in Fig. 4, panel A2. (D) Plate counts of colony morphologies evident in sorted population following plating on MRS agar. (E) Assessment of ^His^GFP-RBP_LMD_ binding to *Ln*. *mesenteroides* (E1 to E3) and L. lactis subsp. *lactis* (E4 to E6) FACS coisolates. Colonies were visualized using fluorescence confocal microscopy at a wavelength of 488 nm (E2 and E4) overlaid on differential interference contrast (DIC) microscopy (E1 and E3). (F) Confirmation of recovery of *Ln*. *mesenteroides* phage LMD_1_2 host by spot assay using original whey sample MUO12, where visible zones of lysis indicate successful infection (F1 to F9).

FACS was then performed in triplicate to isolate labeled cells. Dot plots for one FACS replicate (of a total of three performed) are presented, showing separation of 0.6% GFP-positive cells from the total community (Fig. 4, panel A) compared to 0% for the PB control (Fig. 4, panel B), enabling sorting of the GFP-positive population. This separation was consistent across all replicates with an average of 0.8% (±0.2%) GFP-positive cells. Normalizing processed events across samples to 1,500,000 events resulted in an average of 49,591 ± 19,129 events sorted following incubation with ^His^GFP-RBP_LMD_, while incubation with PB yielded 1.9 ± 0.4 events (Fig. 4, panel C). Plating of sorted events from PB controls yielded no growth, while plating of ^His^GFP-RBP_LMD_ sorted cells on MRS agar yielded colonies with two distinct morphologies, large and small, being present at 6.2 × 10^4^ CFU/mL and 8.67 × 10^3^ CFU/mL across three replicates, respectively (Fig. 4, panel D). 16S rRNA gene sequence analysis of selected cells of the large colony morphology revealed 99% nucleotide identity with strains of *Ln. mesenteroides*, whereas those of the smaller colony morphology exhibited 99% nucleotide identity with L. lactis. This coisolation may be explained by close association of these bacterial genera in milk cultures, as shown and reviewed previously (34, 35). Despite apparent coisolation, binding assays conducted using purified ^His^GFP-RBP_LMD_ and the two bacterial isolates in question showed that ^His^GFP-RBP_LMD_ specifically binds to the *Ln*. *mesenteroides* isolate only (Fig. 4, panels E1 to E3) and not to the L. lactis isolate (Fig. 4, panels E4 to E6).

Having retrieved the presumed *Ln*. *mesenteroides* host from undefined culture, an attempt was then made to retrieve the infecting viral particle. Nine colonies identified as *Leuconostoc* species (three from each replicate) were tested for infection by phages from the original whey sample, using a double layer spot assay method. All selected colonies exhibited sensitivity to phages present in the whey sample, indicated by a zone of clearing on bacterial lawns (Fig. 4, panels F1 to F9). One of these isolates—termed *Leuconostoc* LMDS5—was selected as a host for phage propagation. A single plaque phage isolate from the whey sample was propagated on LMDS5, and DNA from the resulting lysate was isolated. Upon analysis, the genome sequence of the retrieved phage was found to exhibit 100% nucleotide identity with the previously sequenced φLMD_1_2 from the phageome analysis, confirming isolation of a specific phage and host from whey and undefined starter culture, respectively.

To isolate the host of proposed *L*. *cremoris* phage BB4_2 from the second whey sample, *orf04_BB4_2_* was cloned into pHTP9 and an ∼57-kDa protein (^His^GFP-RBP_BB4_2_) was expressed and purified. Fluorescence microscopy revealed specific labeling of a subpopulation of the starter culture, which had been enriched for lactococci by overnight growth in GM17. Following the binding assay, FACS was performed in triplicate to isolate labeled cells. An average separation of 0.11% (±0.07%) GFP-positive cells from the total community was observed. Plating ^His^GFP-RBP_BB4_2_ sorted colonies on GM17 yielded two distinct morphologies, large and small, being present at 1.11 × 10^2^ CFU/mL and 2.43 × 10^2^ CFU/mL, respectively. Interestingly, 16S rRNA sequence analysis revealed isolates of both morphologies belong to closely related *L*. *cremoris*, L. lactis, or Lactococcus laudensis species.

Several lactococcal isolates were selected from the sorted cells for phage sensitivity assays (32). One isolate (termed SC34) exhibited sensitivity to at least one phage present in the whey sample. SC34 was determined by PCR to harbor a B-type CWPS gene cluster, which correlates with the predicted RBP grouping of phage BB4_2, discussed above. A single plaque-purified phage isolate was propagated, and the RBP-encoding gene was amplified and sequenced to confirm 100% nucleotide identity to that assembled from virome data and used for host retrieval.

Interestingly, although *L. cremoris* and L. lactis strains are the most dominant in this mixed starter (accounting for over 95% of reads, based on metagenomic sequencing), CWPS type B strains were not detected during metagenomic read mapping using our method (likely due to insufficient read depth). This indicates that CWPS type B strains were present in very low abundance within the sample. Despite this finding, the presence of strains harboring this gene cluster was in fact detected in the original whey sample by PCR using DNA extracted for metagenome sequencing as the template (32), highlighting the sensitivity of the PhRACS method.

### Phageome analysis ii: Intestinal niche.

To highlight the versatility of PhRACS, it was applied to an alternative ecological niche, i.e., an infant fecal sample. The activities of bifidobacteria and their infecting phages may be a key ecological parameter in the infant gut microbiota, though little is known about their interactions (36–39). Appropriate development of an infant microbiota is linked to positive health outcomes (40–43), and understanding of key modulators of this dynamic ecological environment merits further research.

In order to gain an appreciation of bacterial diversity in the intestinal niche, 16S rRNA gene-encoding sequence analysis was first conducted on a fecal sample (termed T60III) taken from a 30-day-old, vaginally delivered, full-term, breast-fed baby, in order to determine the bacterial component of the microbiota composition (44). Relative abundances of 16S rRNA gene-based sequences detected by phylum, family, and genus are given in Table 3. Bacterial genera detected in this sample included *Bacteroides*, *Parabacteroides*, Escherichia-Shigella, and *Bifidobacterium*. Following this and considering the abundance (Table 3) and relevance of bifidobacteria, internal transcribed spacer (ITS) region sequence analysis was performed to determine the composition of the bifidobacterial population in the sample to species and subspecies level. Species belonging to this genus included Bifidobacterium longum subsp. *longum* (species abundance within genus, 60.45%) and Bifidobacterium pseudocatenulatum (species abundance within genus, 38.51%), with a diverse range of other bifidobacteria also being detected (Table 3).

**TABLE 3:** Microbial composition (greater than or equal to 0.01% read abundance) in fecal sample T60III by phylum, family, genus, and *Bifidobacterium* species

Analysis	Level	Taxon	Abundance (%)
16S	Phylum	*Actinobacteria*	18.78
		*Bacteroidetes*	7.89
		*Firmicutes*	2.26
		*Proteobacteria*	71.05
16S	Family	*Bifidobacteriaceae*	18.72
		*Bacteroidaceae*	6.36
		*Porphyromonadaceae*	1.52
		*Enterobacteriaceae*	71.00
16S	Genus	*Bacteroides*	6.36
		*Parabacteroides*	1.52
		Escherichia *-Shigella*	70.48
		*Bifidobacterium*	18.72
ITS	*Bifidobacterium* species	B. longum subsp. *longum*	60.45
		*B*. *pseudocatenulatum*	38.51
		*B*. *bifidum*	0.89
		*B*. *adolescentis*	0.05
		B. longum subspecies	0.02
		*B*. *parmae*	0.02
		B. longum subsp. *infantis*	0.01
		*B*. *animalis* subsp. *lactis*	0.01
		*B*. *asteroides*	0.01
		*B*. *breve*	0.01
		*B*. *scardovii*	0.01
		Other *Bifidobacterium* species	0.02

To obtain corresponding T60III virome data to enable phage RBP identification, phageome sequencing and analysis of T60III were conducted in parallel. Among 605 (total) phage types whose gene sequences were identified from this analysis was a phage sequence identical to 423phi1, a previously identified inducible prophage of Bifidobacterium breve strain 139W4-23 (39, 44). Following this identification, phageome reads from the T60III fecal sample were mapped against genes encoding 423phi1rv1, the RBP of 423phi1 at a 99% nucleotide identity stringency level (39). This analysis yielded 24 mapped reads, representing a small percentage of the overall number of reads and, indeed, of overall phage diversity in the sample. These results indicated the presence of a phage closely related to 423phi1 and (presumably) a permissive host for that phage in the T60III fecal sample (in agreement with the predicted presence of bifidobacteria), and which we endeavoured to retrieve from this complex sample using PhRACS.

### Host retrieval ii: Intestinal niche.

**FIG 5:**
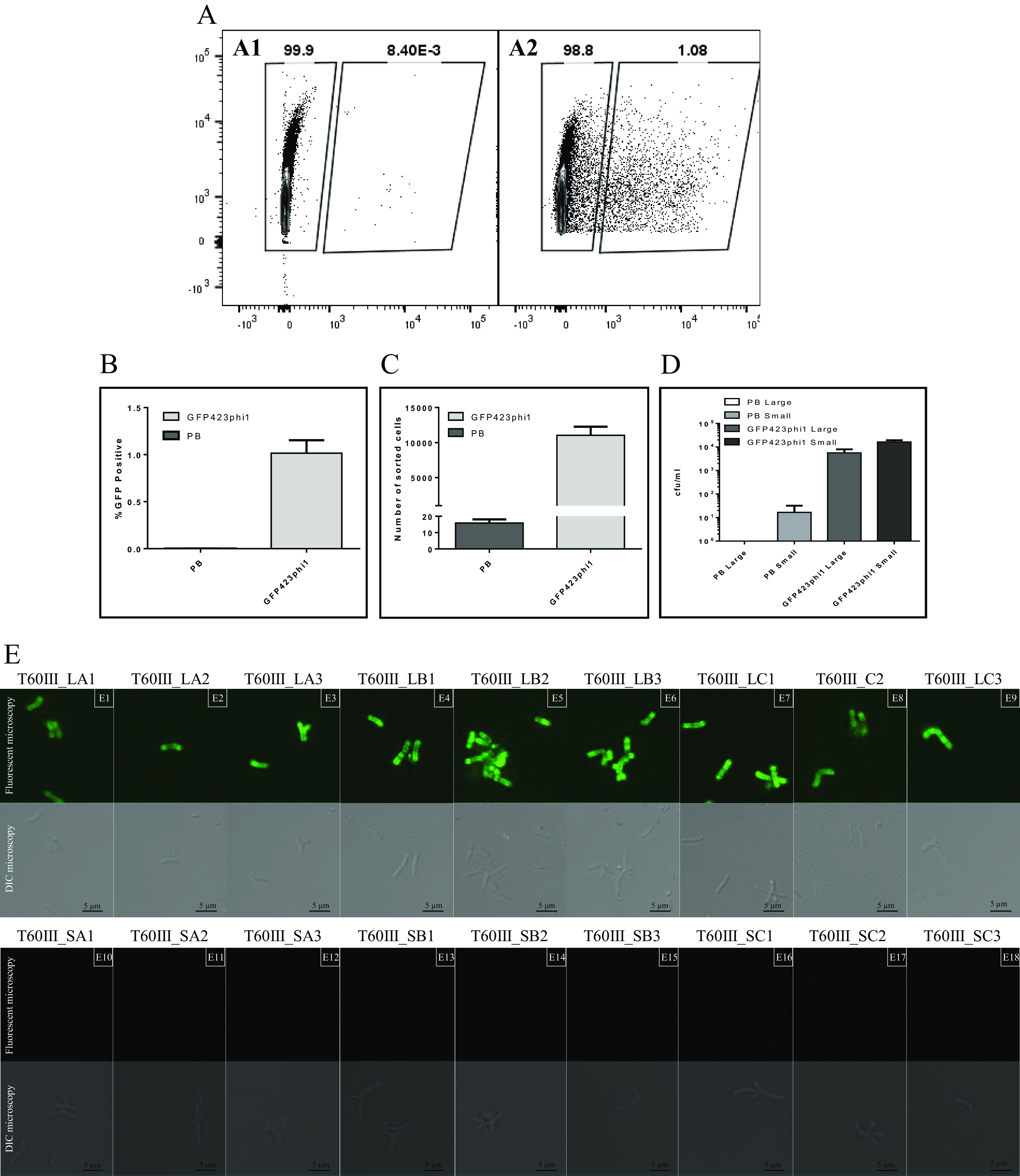
(A) Dot plot analysis of the T60III bifidobacterial community labeled with PB (A1) and ^His^GFP-Rv1_423phi1_ (A2). (B) Proportion of GFP-positive events expressed as a percentage of total events processed. (C) Number of events sorted using gating strategy targeting GFP-positive cells evident in Fig. 4, panel A. (D) Plate counts (on MRS agar) of populations exhibiting distinct colony morphologies present in sorted population. (E) Confirmation of recovery of potential host by GFP-RBP labeling of *B*. *pseudocatenulatum* cultures derived from isolated colonies (E1 to E9). Unlabeled coisolates (B. longum subsp. *longum*) are shown in E10 to E18.

For host retrieval, the bifidobacterial component of sample T60III was cultivated, and the resulting cells were pooled. A GFP-tagged 423phi1Rv1 protein (termed ^His^GFP-Rv1_423phi1_) or PB (as control) was then produced and added to the pooled cells. FACS was then performed in triplicate, and dot plots were generated for one of these replicates in Fig. 5, panel A2. This plot shows separation of 1.08% GFP-positive cells from the total community, compared to 0.008% for the control (Fig. 4, panel A1), enabling sorting of the GFP-positive population. This separation was consistent across all replicates with an average of 1.1% ± 0.14% GFP-positive cells (Fig. 5, panel B).

Normalizing processed events across samples to 1,500,000 resulted in an average of 11,034 ± 1,229 events sorted following incubation with ^His^GFP-Rv1_423phi1_, while incubation with PB yielded 15.87 ± 2.18 events (Fig. 5, panel C). Plating of ^His^GFP-Rv1_423phi1_-sorted cells on RCM agar yielded two colony morphologies, large and small, present at an average of 5.67 × 10^3^ CFU/mL (T60III_L-) and 1.63 × 10^4^ CFU/mL (T60III_S-), respectively, across three replicates (Fig. 5, panel D). In contrast, plating of sorted events from the PB samples yielded an average of 8.33 × 10^0^ colonies.

Eighteen sorted colonies (three representatives of each colony morphology type from each of three replicates) were selected and tested for binding by ^His^GFP-Rv1_423phi1_. Subsequent labeling of isolated colonies revealed that T60III_L-morphotype colonies were labeled (Fig. 5, panels E1 to E9), while all tested T60III_S-morphotype colonies were not labeled by ^His^GFP-Rv1_423phi1_ (Fig. 5, panels E10 to E18). To confirm that isolation of bifidobacterial hosts was achieved, genome sequences of all 18 isolates were determined. *In silico* analysis revealed that all T60III-L isolates were clonal and members of the B. pseudocatenulatum species, while all T60III-S isolates were clonal and members of B. longum subsp. *longum*. The dominance of the nonlabeled strain (B. longum subsp. *longum*) may be due to a physical interaction between labeled and unlabeled cells, since bifidobacteria have previously been shown to auto- and coaggregate in a strain-dependent manner (45–48).

The above demonstrates enrichment of the ^His^GFP-Rv1_423phi1_ binding population from 1.1% to 34.8% (Fig. 5, panel D) and the ability to isolate pure culture of this ^His^GFP-Rv1_423phi1_-interacting population. Current laboratory conditions are not permissive of plaque formation using bifidobacteria (39), limiting additional analyses of this bifidobacterial phage-host interaction.

### Concluding remarks.

Here, we established a method enabling isolation of phage-host combinations from mixed, complex bacterial communities using FACS, where hosts are labeled using probes derived from the cell-surface recognition protein of its infecting phage.

To infect its host, a bacteriophage must specifically bind to a molecule that is exposed at the host cell surface, representing the first stage of infection (12). As such, PhRACS relies on prediction of a phage-encoded RBP by phageome analysis, followed by construction of a GFP-RBP fusion protein which allows for specific labeling of its putative host within a complex community. These labeled hosts are then isolated by fluorescence-mediated cell sorting, as previously seen in methods such as viral tagging (11). A major advantage of PhRACS over similar methods is that it allows isolation of a host-phage combination from a given environmental sample using just phageome sequence data as a starting point. In contrast, other methods (such as viral tagging) allow only isolation of phages infecting a known host.

To demonstrate its utility, proof of concept was first established, and PhRACS was then applied to two complex samples, i.e., a whey sample derived from a fermentation utilizing an undefined mesophilic culture (MUO12), and an infant fecal sample (T60III). Following identification of a putative *Leuconostoc* phage RBP using phageome analysis of MUO12, construction of a GFP-fusion protein and PhRACS application resulted in an enrichment of the target population from 0.6% to 87%, enabling straightforward host isolation by routine methods.

Using a fluorescently tagged RBP of a previously described bifidophage, we successfully isolated a fecal sample-derived bifidobacterial strain, to which this RBP specifically binds. Due to the apparent recalcitrance of *Bifidobacterium* phages to form *in vitro* plaques, further study of the interaction is currently difficult. Nonetheless, this method represents an ideal tool for further exploration of these interactions with the capacity to prove both binding activity of a putative *Bifidobacterium* (or, indeed, any) phage and isolation/identification of potential hosts.

At present, several limitations of the current method can be identified. First, the RBP-encoding gene of the target phage must be predicted *in silico* to produce a suitable “hook” with which to retrieve the phage host. This may be an issue in cases where phages do not encode an easily recognizable RBP-encoding gene. To overcome this (at least for phages binding to carbohydrate receptors), identified carbohydrate binding domains (CBMs), responsible for phage host adhesion (49) and which have been shown to be host specific (50, 51), could be employed.

Second, retrieval of two distinct bacterial isolates using a single phage RBP hook occurred during whey and infant fecal sample processing. While the reason for this remains unclear, we postulate that this is due to close association of coisolated strains in their natural environment, either in biofilms or through general aggregation. Another cause may be the use of an 85-μm nozzle during cell sorting (see Materials and Methods), which was employed to decrease cell stress and improve purity. Future studies may benefit from the use of a smaller nozzle, or indeed single cell sorting, as has been employed in other studies (52). In addition, physical or chemical pretreatment of samples may be necessary to disrupt biofilms prior to performing binding assays. Third, in both examples presented above (dairy and intestinal niches), target bacterial populations were enriched by cultivation prior to GFP-RBP binding to increase the likelihood of host identification. Further studies are required to investigate whether this cultivation is necessary, which may provide further evidence of the robustness of PhRACS.

Beyond isolation of hosts for phages, this protocol has further applications that could be investigated. The advent of metagenomics has resulted in a large-scale exploration of diverse and microbe-rich communities, including soil (53), marine (54, 55), and wastewater-related sites (56, 57) and the gut (43). Considering that prophages are widely distributed on bacterial genomes sequenced from these environments and that the prophage must have adsorbed to the host surface through a cell-surface receptor, a recombinant phage RBP may act as a hook by which bacterial strains of interest may be isolated. Theoretically, any prophage-harboring bacterial strain may be isolated from a complex community using this technique. In the event that a host is not isolated, enrichment of the RBP-bound bacterial host may be sufficient to enable determination of its genome sequence, by comparing metagenome data from the original sample to that of a sample having undergone PhRACS. Further applications of PhRACS (or variations thereof) will undoubtedly be identified in future attempts to bridge the current gap between metagenomics and more traditional culture-based microbiology.

## MATERIALS AND METHODS

### Bacterial and phage culture conditions and storage.

Phages, bacterial cultures, and plasmids employed in this study are listed in Table 1. Standard bacterial and phage culture conditions (see supplementary method 1 in Text S1 in the supplemental material) were employed.

10.1128/mbio.03334-21.2 TEXT S1 Supplementary methods containing nine supplementary methods and supplementary References. The supplementary methods are as follows: supplementary method 1, bacterial and phage culture and storage conditions; supplementary method 2, molecular cloning; supplementary method 3, recombinant protein production; supplementary method 4, binding assays; supplementary method 5, metavirome analysis; supplementary method 6, metagenome extraction and analysis; supplementary method 7, fecal sample processing; supplementary method 8, genome assembly and annotation; supplementary method 9,16S rRNA and ITS sequence-based microbiota determination. Download Text S1, DOCX file, 0.1 MB. Copyright © 2022 Casey et al. 2022 Casey et al. https://creativecommons.org/licenses/by/4.0/ This content is distributed under the terms of the Creative Commons Attribution 4.0 International license.

### Molecular cloning.

Primers used in this study (Table 2) were synthesized by Eurofins Genomics (Germany). PCR amplifications were performed using Phusion High Fidelity DNA polymerase (New England Biolabs, UK). Restriction enzymes were purchased from Roche (Germany), and T4 DNA ligase was obtained from Promega (USA). All restriction digestions and ligations were performed using standard molecular cloning techniques (58) employing restriction enzymes (where appropriate) specified in Table 2 and as described in supplementary method 2 in Text S1. Recombinant protein production is described in supplementary method 3 in Text S1.

### Binding assays.

Qualitative assessment of the binding of ^GFP^RBP fusion proteins to bacterial cell surfaces was performed by a method adapted from Dieterle et al. (19) and as described in supplementary method 4 in Text S1. Fluorescence microscopy was performed using a Zeiss LSM 5 Exciter (Carl Zeiss, Germany) utilizing an argon laser and detector and filter sets for monitoring GFP (excitation, 488 nm; emission, 505 to 550 nm).

### Flow cytometry cell preparation.

For assessment using ^GFP^UAL_Tuc2009_, 150 μL of early exponential phase (optical density at 600 nm [OD_600_] ≈ 0.2) of L. lactis UC509.9::pNZ44 cells was harvested, resuspended in 150 μL of SM buffer, and mixed with similar phase L. lactis 3107 wild type. Plate counts of both strains were undertaken at the point of harvesting prior to binding assays.

For assessment using ^GFP^UL_TP901-1_, 150 μL of exponential-phase L. lactis 3107-pNZ44 was harvested and resuspended in 150 μL of SM buffer. A serial dilution was made in SM buffer to a theoretical CFU/mL of 1. To do this, 150 μL of undiluted resuspended L. lactis UC509.9 wild type and 150 μL of each serial dilution were mixed to generate eight samples with a sequential 10-fold reduction in target concentration and with a constant nontarget cell concentration. Binding assays were then performed as described above.

In the case of ^GFP^RBP_LMD_, bulk starter culture was inoculated into GM17 and MRS broth separately and incubated at 30°C until it reached an OD_600_ ≈ 0.2 to 0.3. Cells were harvested, resuspended in SM buffer, and mixed in equal volumes to 300 μl prior to binding assays. In the case of ^GFP^RBP_BB4_2_, bulk starter was cultured in GM17 as described above.

In the case of ^GFP^Rv1_423phi1_, the presumed bifidobacterial component (having been enriched as described) of the T60III fecal sample was cultured in RCM as described above until it reached an OD_600_ ≈ 0.2 before binding assays.

### Flow cytometry.

GFP-RBP fusion protein-labeled bacteria were analyzed and sorted on a BD ARIA Fusion cell sorter using 488-nm laser for GFP excitation and 530/30 band pass detector filter. To minimize electronic noise and detect relatively small bacterial cells, adjustments were made as follows: side scatter and forward scatter signals were acquired with amplifiers set to logarithmic scale, and threshold level was set for side scatter and adjusted to minimize noise as much as possible. Samples were sorted with 85-μm nozzle, fluidics pressure at 47 lb/in^2^ using drop delay of 31.04 μs, and DIVA software sorting precision protocol 16-16-0. One hundred thousand events were recorded per sample. Data analysis was performed using FlowJo (59).

### Metavirome extraction, sequencing, and analysis.

Whey phageome sequencing was performed as follows. NaCl was added to 1 ml of whey sample (to a final concentration of 1 M) which was incubated at 4°C for at least 1 h. The pH was adjusted to 4.6 to clarify, and the solution was centrifuged for 15 min at 28,000 × *g*. Polyethylene glycol 8000 (PEG8000) (Sigma-Aldrich) was added to a final concentration of 10% (wt/vol), and supernatant was incubated on ice for 1 h. PEG-precipitated phages were harvested by centrifugation at 15,000 × *g* for 15 min and resuspended in 1 ml of SM buffer. DNase treatment and viral DNA extraction were as previously described (5). DNA preparation, sequencing, assembly, and read mapping and retrieval were as previously described (5). Gene products of LMD_1_2 were interrogated for similarity to RBPs using HHPred (26) and BLASTP (60), while putative host species for phage contigs were predicted using HostPhinder (9). Phageome sequencing of infant fecal samples was performed as previously described (5). Quality filtering and classification of reads were performed as described in supplementary method 5 in Text S1.

### Fecal sample collection and processing.

A fecal sample was collected from a 30-day-old, full-term, breast-fed infant. A fecal sample was collected in a sterile container, immediately frozen at −20°C prior to processing (see supplementary method 5 in Text S1). The study was approved by the Regional Ethical Committee of Asturias Public Health Service (reference 12/16), and informed written consent was obtained from the parents.

### Genome sequencing.

Genome sequences of 18 *Bifidobacterium* isolates from the T60III sample were determined as previously described (61) using a Illumina NextSeq 550 platform with NextSeq 500/550 v2.5 High Output kit v2.5 300 cycles, and assembled and annotated according to supplementary method 6 in Text S1.

### 16S rRNA and ITS sequence-based microbiota determination.

Bacterial DNA was used as the template for partial 16S rRNA gene PCR amplification, followed by Illumina MiSeq sequencing as previously described (62). Presumed bifidobacterial DNA (extracted from the enriched bifidobacterial portion of the T60III fecal sample; see supplementary method 7 in Text S1) was used as the template for partial ITS PCR amplification, followed by Illumina MiSeq sequencing as previously described (37, 63).
