# Supplementary material for: Needle in a Whey-Stack: PhRACS as a Discovery Tool for Unknown Phage-Host Combinations
Source: mBio. 2022 Jan 4;13(1):e03334-21. doi: 10.1128/mbio.03334-21 (PMC8725590; doi:10.1128/mbio.03334-21)
Supplement: TEXT S1 [file mbio.03334-21-s0001.docx]

**Supplementary Method 1.** Bacterial and phage culture and storage conditions

*L*. *lactis* strains were cultivated in GM17 (M17 Broth [Oxoid, UK] supplemented with 0.5 % glucose [Sigma, UK]) at 30°C, supplemented with 5 μg/ml chloramphenicol where appropriate. *Leuconostoc* strains were cultivated in MRS broth (Difco, BD, France) at 28°C. Bifidobacteria were routinely cultured in pre-filtered Reinforced Clostridial Medium (RCM; Oxoid) or Brain Heart Infusion broth (BHI; Oxoid) at 37°C in a modular atmosphere (10% H_2_, 10% CO_2_, 80% N_2_) controlled system (Davidson and Hardy, Belfast, Ireland). Bulk starter culture was produced by incubating lyophilised commercial starter culture in either 10 % (w/v) reconstituted skim milk powder (Carbery Ingredients, Ireland) (for use with ^His^GFP-RBP_LMD_) or GM17 (for ^His^GFP-RBP_BB4_2_) and incubating at 30°C or 22℃ for 18 hours, respectively. Where employed, pooled or complex cultures were generated by growing two or more strains individually in applicable medium to OD_600nm_ ≈ 0.2 - 0.3, prior to combining equal volumes of each. Phages were propagated and lysates stored essentially as previously described [1].

**Supplementary Method 2.** Molecular cloning

pNZ8048-His-GFP-UALTuc2009 (encoding an N-terminally His-tagged GFP fused to the N-terminus of Tuc2009 tripod complex) and pNZ8048-His-GFP-ULTP901-1 (encoding a similarly tagged TP901-1 tripod complex) were constructed as follows. GFP-UALTuc2009 and GFP-ULTP901-1 specifying amplicons were firstly generated using a splicing by overlap extension (SOE) strategy. Primers GFPFwHis and GFPRevlinkBppU (Table 2) were used to amplify the GFP-encoding gene from pZEP08 [2], while primer combination BppUF and BppLTucR or BppLTPR were used to amplify the coding region of tripod complexes of Tuc2009 and TP901-1, respectively. These amplicons contained complementary overhangs that were utilised as a combined template to generate a GFP-UAL_Tuc2009_ and GFP-UL_TP901-1_-specifying DNA regions, and subsequently amplified using primer pair GFPFwHis and BppLTucR or BppLTPR, respectively. These amplicons were digested and ligated to pNZ8048 and introduced to *L*. *lactis* NZ9000 as described by Holo et al. [3].

To facilitate routine cloning of putative RBP-encoding genes, a pNZ8048 [4] -based GFP-fusion vector, pGFP8048 was constructed as follows: the DNA fragment containing the *gfp*+ gene was amplified from the pZEP08 plasmid [2], and flanked with a poly-histidine tag and a flexible linker sequence using primers GFP8048F and GFP8048R (Table 2).

pGFP8048- His-GFP-RBPLMD (encoding an N-terminally His-tagged GFP fused to the N-terminus of the ΦLMD_1_2 RBP) was constructed as follows: primer pair LMD22F and LMD22R (Table 2) were used to amplify orf22LMD_1_2 using whey sample phageome DNA as template. The resultant amplicon was ligated to pNZ8048 and introduced to NZ9000 as described above.

pHTP9-423phi1Rv1 was constructed as follows: prophage 423phi1 was induced from its host using mitomycin C as previously described [5]. DNA was extracted from the resultant filtered lysate for subsequent use as template DNA for gene amplification. The purified Rv1423phi1 PCR product was then cloned into GFP fusion vector pHTP9 (GFP) using the NZYEasy Cloning Kit (NZYTech genes & enzymes, Portugal) as per the manufacturer’s instructions, and introduced to competent *E*. *coli* BL21 (DE3) by heat shock transformation [6].

pHTP9-RBP_BB4_2_ was constructed as follows: primer pair BB4-RBPF and BB4-RBPR (Table 2) were used to amplify BB4_2*_orf04_* using whey sample phageome DNA as template. The purified BB4_2*_orf04_* product was cloned into GFP fusion vector pHTP9 as described above.

## **Supplementary Method 3.** Recombinant Protein Production

Protein expression from pNZ8048, extraction and visualisation were carried out as previously described (16), with the exception of the duration of the ice incubation during sonication, which was shortened to 30 sec. His-tagged proteins were purified using a Ni-nitrilotriacetic acid (NTA) agarose (Qiagen, UK) with samples eluted (1 ml fractions) in elution buffer (10 mM Tris, 300 mM NaCl, 20 mM CaCl_2_ [pH 8]) with increasing concentrations of imidazole (50 – 250 mM) followed by (where necessary) size exclusion chromatography on a Superdex 200 10/300 GL column (GE Healthcare Life Sciences, USA) in 50 mM Tris-HCl containing 300 mM NaCl and 50 mM CaCl_2_ (pH 7.4).

Protein expression from pHTP9 was carried out as follows: 1 ml aliquots of *E. coli* BL21 (DE3) carrying recombinant plasmids were inoculated in 100 mL of auto-induction medium (NZYTech genes and enzymes, Portugal) supplemented with 50 μg/ml of kanamycin followed by incubation at 24 °C for 24 hours in a shaking incubator at 300 rpm. Cells were harvested by centrifugation at 4000 x *g* for 30 mins and resuspended in 20 mL of lysis buffer (50 mM Tris pH 8.0, 500 mM NaCl, 5% glycerol, 1% triton-X100, 30 mM imidazole, 50 mg lysozyme), and frozen at -80 °C overnight. The cells were then disrupted by sonication in an MSE Soniprep (Sanyo, Japan) at 20 μm for five 30 s cycles separated by 30 s cooling on ice. Subsequent centrifugation at 25,000 x *g* separated cell debris and insoluble fractions from the sample. Target proteins were then purified on an Ni-NTA agarose column (Qiagen, Manchester, UK) as described above. Purified proteins were then dialysed against protein storage buffer (50mM Tris-HCl, 300 mM NaCl, 50 mM CaCl_2_, pH 8.0) and stored at 4°C.

**Supplementary Method 4.** Binding assays

For binding to *L*. *lactis*, 300 μL of exponential phase culture was harvested and resuspended in 300 μL SM Buffer (50 mM Tris-HCl, 100 mM NaCl, 10 mM MgSO_4_, 10 mM CaCl_2_). 25 μg of fusion protein was incubated with the resuspension or growing culture at 30°C for 12.5 min. For binding to bifidobacteria and *Leuconostoc*, 300 μL of exponential phase culture grown in pre-filtered RCM (Oxoid Ltd., Basingstoke, England) or MRS was incubated with 25 μg of each GFP fused RBP at 37°C or 30°C respectively for 12.5 min. In all cases, the mixtures were washed three times with phosphate-buffered saline (PBS) buffer prior to imaging of protein-cell complexes, which was performed by fluorescent confocal microscopy. In the case of ^GFP^RBP_LMD_, bulk starter culture was inoculated into GM17 and MRS broth separately, incubated at 30°C until OD_600nm_ ~ 0.2, harvested, and resuspended in SM buffer prior to combining 150 µL of each. In the case of ^GFP^RBP_BB4_2_, bulk starter culture was propagated in GM17 only. Binding of the fusion protein was then performed as described above.

**Supplementary Method 5.** Metavirome analysis

Quality filtering of each individual sample was improved with a ﬁltering step performed to obtain only high-quality reads (minimum mean quality score 20, window size 5, quality threshold 25 and minimum length 80) using the fastq-mcf script (https://github.com/ExpressionAnalysis/ea-utils/blob/wiki/FastqMcf.md). Collected filtered reads were assembled using SPAdes v3.13.0 [7] and the resulting contigs were taxonomically classified based on homology searches deﬁned by means of RAPSearch2 (Reduced Alphabet based Protein similarity Search 2) [8] based on RefSeq NCBI databases. Above-mentioned steps, as well as the taxonomic classification of the filtered paired-end reads were independently performed by the METAnnotatorX pipeline [9].

**Supplementary Method 6.** Metagenome extraction and analysis

Bulk starter culture was propagated by first inoculating lyophilised commercial starter culture in sterilized 10 % (w/v) reconstituted skim milk powder (Carbery Ingredients, Ireland) and incubating at 22℃ for 18 hours. This was followed by a second inoculation of the culture at 3% (v/v) in pasteurized 10 % (w/v) reconstituted skim milk powder (Carbery Ingredients, Ireland). The metagenomic DNA was then extracted as described by Erkus et al. [10]. The relative abundance of each CWPS cluster was determined through metagenomic reads mapping using the alignment tool bowtie2 [11]. Filtered raw metagenomic reads were mapped against conserved ORFs representing different CWPS biosynthesis gene clusters: *UC509_0206* (CWPS type A),); *LLKf_0205* (B), *llmg_0220* (C_1_), ; *L3107_0192* (C_2_),; *3107_004*; *LACR_0215* (C_3_),; *w34_004* (C_4_),; *lilo_0174* (C_5_), *llh_1295* (C_6_), and *LL184_0294* (D). Primers specific to the A-C CWPS gene clusters, previously described by Mahony et al. [12], were also used to determine if any of the CWPS gene clusters were present in the bulk starter culture using the metagenomic DNA as template.

**Supplementary Method 7.** Fecal sample processing

0.5 g of faecal sample was resuspended 5 ml PBS (0.05 % cysteine-HCl (w/v)) homogenised by vortexing. 500 μl of this suspension was spread plated across four plates of RCM agar (Oxoid) supplemented with 0.5 % lactose, 1 mM ferulic Acid, 5μg/ml hemin, 100 μg/ml mupirocin and 50 μg/ml nystatin followed by incubation for 48 hours at 37°C in a modular atmosphere controlled system (as above). The resulting colonies/lawn was resuspended in PBS (0.05 % cysteine-HCl) using disposable scrapers and stored in 20 % glycerol at -80°C. This mixture was then used as the inoculum for labelling and FACS as described above.

For bacterial DNA isolation from faeces, 0.5 g of faecal sample was diluted in 5 ml sterile PBS and homogenized in a Lab-Blender 400 stomacher (Seward Medical, London, UK) for 5 min. Cells were separated from 1 ml of homogenized sample by centrifugation and bacterial DNA isolated using the QIAmp DNA stool kit (Qiagen, Hilden, Germany) following the manufacturer’s instructions.

**Supplementary Method 8.** Genome assembly and annotation

Genomes were assembled from raw reads using SPAdes (v 3.11.1) [7] with kmer lengths of 33,55,77,99 and 127. Contigs shorter than 1000 bp were excluded and coverage was calculated by aligning raw reads to the assembled contigs using Bowtie 2 (v 2.2.7) [11]. Genomes were annotated as follows: Open reading frames were predicted with Prodigal [13] in anon mode (-p anon). Functions of protein coding sequences were annotated using a combination of BLAST (blastp v2.2.28+) [14] against an in-house bifidobacterial database and HMMER (v 3.1b1) [15] against the Pfam [16] database. Transfer ribonucleic acids (tRNAs) were predicted using tRNA-scan-SE (v 1.3.1) [17].

**Supplementary Method 9.** 16S rRNA and ITS sequence-based microbiota determination

Individual reads were filtered, trimmed and processed following a custom bash script based on the QIIME software suite [18]. Then, 16S rRNA Operational Taxonomic Units (OTUs) were defined at 97 % sequence homology using the UCLUST tool [19] and classified from phylum down to the genus level by means of the SILVA database v.123 [20]. Relative abundances were calculated based on the total number of reads.

Following sequencing of presumed bifidobacterial DNA for ITS sequence based microbiota determination, the .fastq files were processed using a custom script based on the QIIME2 software suite [18]. Quality control retained sequences with a length between 100 and 400 bp and mean sequence quality score of >20, while sequences with homopolymers >7 bp in length and mismatched primers were removed. ITS Operational Taxonomic Units (OTUs) were defined at 100 % sequence homology using DADA2 [21]. All reads were classified to the lowest possible taxonomic rank using QIIME2 [22] and a reference dataset, consisting of an updated version of the bifidobacterial ITS database [23]. Relative abundances were calculated based on the total number of reads.

**Supplementary References**

1. Mahony J, Tremblay DM, Labrie SJ, Moineau S, van Sinderen D. Investigating the requirement for calcium during lactococcal phage infection. *Int J Food Microbiol* 2015; **201**: 47–51.

2. Hautefort I, Proença MJ, Hinton JCD. Single-copy green fluorescent protein gene fusions allow accurate measurement of *Salmonella* gene expression in vitro and during infection of mammalian cells. *Appl Environ Microbiol* 2003; **69**: 7480–7491.

3. Holo H, Nes IF. High-frequency transformation, by electroporation, of *Lactococcus lactis* subsp. *cremoris* grown with glycine in osmotically stabilized media. *Appl Environ Microbiol* 1989; **55**: 3119–3123.

4. Kuipers OP, de Ruyter PGGA, Kleerebezem M, de Vos WM. Quorum sensing-controlled gene expression in lactic acid bacteria. *J Biotechnol* 1998; **64**: 15–21.

5. Mavrich TN, Casey E, Oliveira J, Bottacini F, James K, Franz C, et al. Characterization and induction of prophages in human gut-associated *Bifidobacterium* hosts. *Sci Rep* 2018; **8**: 12772.

6. Van Die IM, Bergmans HEN, Hoekstra WPM. Transformation in *Escherichia coli*: studies on the role of the heat shock in induction of competence. *Microbiology* 1983; **129**: 663–670.

7. Bankevich A, Nurk S, Antipov D, Gurevich AA, Dvorkin M, Kulikov AS, et al. SPAdes: a new genome assembly algorithm and its applications to single-cell sequencing. *J Comput Biol* 2012; **19**: 455–477.

8. Zhao Y, Tang H, Ye Y. RAPSearch2: a fast and memory-efficient protein similarity search tool for next-generation sequencing data. *Bioinformatics* 2012; **28**: 125–126.

9. Milani C, Casey E, Lugli GA, Moore R, Kaczorowska J, Feehily C, et al. Tracing mother-infant transmission of bacteriophages by means of a novel analytical tool for shotgun metagenomic datasets: METAnnotatorX. *Microbiome* 2018; **6**: 145.

10. Erkus O, De Jager VCL, Spus M, Van Alen-Boerrigter IJ, Van Rijswijck IMH, Hazelwood L, et al. Multifactorial diversity sustains microbial community stability. *ISME J* 2013; **7**: 2126–2136.

11. Langmead B, Salzberg SL. Fast gapped-read alignment with Bowtie 2. *Nat Methods* 2012; **9**: 357–359.

12. Mahony J, Kot W, Murphy J, Ainsworth S, Neve H, Hansen LH, et al. Investigation of the relationship between lactococcal host cell wall polysaccharide genotype and 936 phage receptor binding protein phylogeny. *Appl Environ Microbiol* 2013; **79**: 4385–4392.

13. Hyatt D, Chen G-L, LoCascio PF, Land ML, Larimer FW, Hauser LJ. Prodigal: prokaryotic gene recognition and translation initiation site identification. *BMC Bioinformatics* 2010; **11**: 119.

14. Altschul SF, Gish W, Miller W, Myers EW, Lipman DJ. Basic local alignment search tool. *J Mol Biol* 1990; **215**: 403–410.

15. Finn RD, Clements J, Eddy SR. HMMER web server: interactive sequence similarity searching. *Nucleic Acids Res* 2011; **39**: W29–W37.

16. El-Gebali S, Mistry J, Bateman A, Eddy SR, Luciani A, Potter SC, et al. The Pfam protein families database in 2019. *Nucleic Acids Res* 2018; **47**: D427–D432.

17. Chan PP, Lowe TM. tRNAscan-SE: searching for tRNA genes in genomic sequences. *Gene Prediction*. 2019. Springer, pp 1–14.

18. Caporaso JG, Kuczynski J, Stombaugh J, Bittinger K, Bushman FD, Costello EK, et al. QIIME allows analysis of high-throughput community sequencing data. *Nat Methods* 2010; **7**: 335–336.

19. Edgar RC. Search and clustering orders of magnitude faster than BLAST. *Bioinformatics* 2010; **26**: 2460–2461.

20. Quast C, Pruesse E, Yilmaz P, Gerken J, Schweer T, Yarza P, et al. The SILVA ribosomal RNA gene database project: improved data processing and web-based tools. *Nucleic Acids Res* 2013; **41**: D590-6.

21. Callahan BJ, McMurdie PJ, Rosen MJ, Han AW, Johnson AJ, Holmes SP. DADA2: High-resolution sample inference from Illumina amplicon data. *Nat Methods* 2016; **13**: 581–583.

22. Bokulich NA, Kaehler BD, Rideout JR, Dillon M, Bolyen E, Knight R, et al. Optimizing taxonomic classification of marker-gene amplicon sequences with QIIME 2’s q2-feature-classifier plugin. *Microbiome* 2018; **6**: 90.

23. Milani C, Lugli GA, Turroni F, Mancabelli L, Duranti S, Viappiani A, et al. Evaluation of bifidobacterial community composition in the human gut by means of a targeted amplicon sequencing (ITS) protocol. *FEMS Microbiol Ecol* 2014; **90**: 493–503.
